# Analog monolayer SWCNTs-based memristive 2D structure for energy-efficient deep learning in spiking neural networks

**DOI:** 10.1038/s41598-023-48529-z

**Published:** 2023-12-04

**Authors:** Heba Abunahla, Yawar Abbas, Anteneh Gebregiorgis, Waqas Waheed, Baker Mohammad, Said Hamdioui, Anas Alazzam, Moh’d Rezeq

**Affiliations:** 1 Quantum & Computer Engineering Department, Delft University of Technology, Delft, The Netherlands; 2 System on Chip Center (SoCC), Physics Department, Khalifa University, Abu Dhabi, UAE; 3 SoCC, Mechanical Engineering Department, Khalifa University, Abu Dhabi, UAE; 4 SoCC, Electrical Engineering & Computer Science Department, Khalifa University, Abu Dhabi, UAE

**Keywords:** Engineering, Materials science, Nanoscience and technology

## Abstract

Advances in materials science and memory devices work in tandem for the evolution of Artificial Intelligence systems. Energy-efficient computation is the ultimate goal of emerging memristor technology, in which the storage and computation can be done in the same memory crossbar. In this work, an analog memristor device is fabricated utilizing the unique characteristics of single-wall carbon nanotubes (SWCNTs) to act as the switching medium of the device. Via the planar structure, the memristor device exhibits analog switching ability with high state stability. The device’s conductance and capacitance can be tuned simultaneously, increasing the device's potential and broadening its applications' horizons. The multi-state storage capability and long-term memory are the key factors that make the device a promising candidate for bio-inspired computing applications. As a demonstrator, the fabricated memristor is deployed in spiking neural networks (SNN) to exploit its analog switching feature for energy-efficient classification operation. Results reveal that the computation-in-memory implementation performs Vector Matrix Multiplication with 95% inference accuracy and few femtojoules per spike energy efficiency. The memristor device presented in this work opens new insights towards utilizing the outstanding features of SWCNTs for efficient analog computation in deep learning systems.

## Introduction

Building ultra-low-power computing systems is a key driver for the rise and innovation of artificial intelligence (AI), particularly deep learning (DL)^1^. Bio-inspired (neuromorphic) DL algorithms are mostly implemented using conventional Von Neumann computing systems, in which memory and computation are performed in separate units; this substantially degrades the system’s efficiency and overall performance. Thus, great research interest has been drawn towards utilizing a new class of non-volatile memory devices (namely, memristor) to achieve brain-inspired computing, where storage and computation can be achieved in the same memory crossbar^2^. The main biological behaviors needed to be accommodated by the memory devices are synaptic plasticity and synaptic efficacy^3^. Synaptic plasticity is associated with the ability of the device to change its resistance state under the application of sufficient writing voltage, while synaptic efficacy is the ability to read the state of the memory device with the applied reading voltage. In this context, exploring new materials to manufacture appropriate new non-volatile memory devices and being able to accurately mimic the biological behavior of the brain is of great importance.

Nanomaterials have inspired researchers due to their unique nanoscale size-dependent characteristics compared to their bulk counterparts^4–6^. Several bio-inspired memristor devices have been reported in the literature using various material combinations and following different switching mechanisms^7–9^. Recently, great interest has been directed toward utilizing the unique characteristics of carbon-related materials, such as graphene oxide (GO) and carbon nanotubes (CNTs) to achieve energy-efficient memristor devices^10,11^. In GO-based memristors, the resistance switching is mainly caused by the defective nature of GO film which limits its practical application^12^. Moreover, tuning the conductivity of GO according to the target switching behavior can be achieved via the reduction methodologies available in the literature (i.e. thermal, electrical, and chemical reduction)^13^. This adds to the complexity of the fabrication process and limits the selection of the electrodes and substrate materials according to the reduction methodology. On the other hand, CNTs can provide higher thermal stability^11^ and their conductivity can be controlled via careful optimization of their concentration in the used solvent. Also, CNTs provide the ability to diminish the memory cell diameter to ~ 2 nm^14^. Thus, recently, researchers have been interested in deploying CNTs as a switching medium for non-volatile memristor devices. The work in Ref.^14^ presents a memristor device fabricated by growing vertically aligned CNTs on a Ni/Ti/Si substrate. Memristors based on strained multi-walled CNTs (MWCNTs) have been reported in Ref.^12^. The switching characteristics of the devices are explored under atmospheric and vacuum conditions and it’s shown that the OFF/ON resistance ratio is increased up to 10 times under vacuum. Memristor devices based on MWCNTs and Graphene Quantum Dots (GQD) are provided in Ref.^15^. The devices exhibit ultraviolet (UV) light sensitivity due to the use of GQD and it’s been proved that the switching current ratio of the device can be controlled by tuning the content of GQDs in the dielectric layer and the UV illumination duration. Authors in Ref.^16^ propose embedding single-wall CNTs (SWCNTs) in the switching medium of Ti/SiO_2_/Pt/Ti/ITO which improves the switching window of the memristor device. CNTs decorated with gold nanoislands are used as the switching film in the memristor device reported in Ref.^11^. The switching mechanism is shown to be based on trapping thermionic emission of the electrons in the nanoislands. All the devices reported in Refs.^11,12,14–16^ exhibit binary switching behavior which constrains the device's applications, as multistate switching is considered a preferable characteristic for neuromorphic computing applications. Hence, there is a need to explore new device structures and material combinations toward achieving enhanced memory characteristics to accommodate the energy and accuracy requirements of bio-inspired DL systems.

This work advances the state of the art by developing, designing, and manufacturing an analog planar memristor device. The device is fabricated using a monolayer of single-wall CNTs (SWCNTs) deposited between two gold electrodes on a flexible substrate. Experimental results show multi-state storage capability and long-term memory characteristics of the device, which make it a potential candidate for energy-efficient computing. To prove this, the fabricated memristor device is utilized to perform classification in spiking neural networks based on its analog switching ability. Results are promising as energy-efficient Vector Matrix Multiplication (VMM) is achieved with 95% inference accuracy.

## Materials and methods

**Figure 1:**
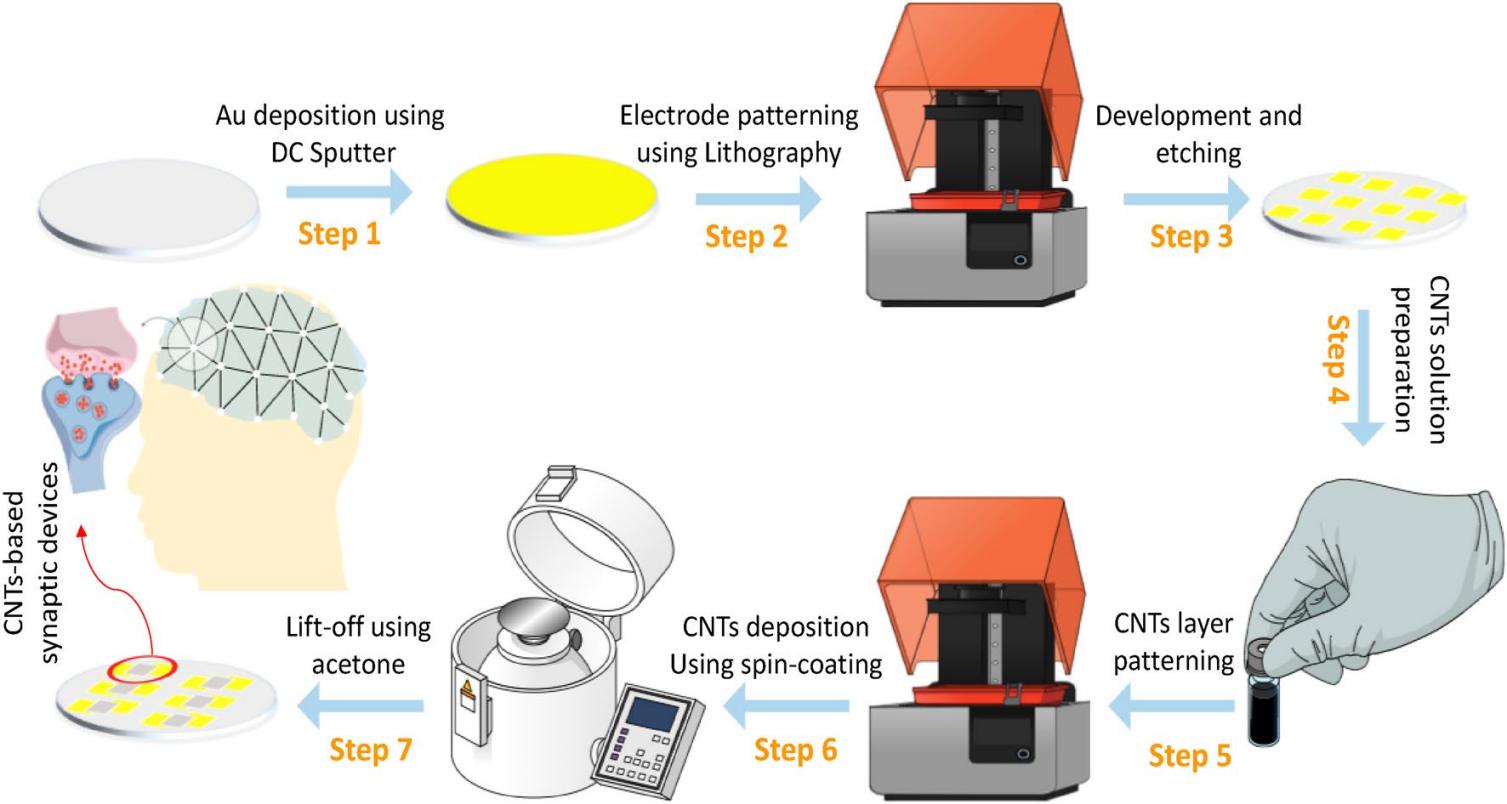
Schematic diagram of the fabrication steps of the device. This includes patterning the gold electrodes using Etching/Lithography process and patterning the SWCNTs-based active layer using Lift-off/Lithography process. Drawings are prepared using *Mindthegraph* software and *PowerPoint* drawing tools.

Figure 1 provides a schematic of the fabrication steps followed to achieve the planar synaptic cells reported in this work. In Step 1, Au deposition is performed on a flexible Cyclic Olefin Copolymer (COC) substrate using DC sputtering. After that, in Step 2, a photoresist layer is spin-coated and the electrode patterns are printed using a photolithography system. Then, development and etching (using Au etchant) processes are followed to achieve the Au electrodes as described in Step 3. In Step 4, the SWCNTs solution is prepared by dissolving 1.2 ml of SWCNTs (purity > 95%, ignited temperature > 610 degrees, dispersed in water) in 14 ml of DI water. This has been decided via a careful optimization process to achieve the target device characteristics. The prepared solution is sonicated for 2 min. Later, a lift-off-based lithography process is used to pattern the SWCNTs monolayer between the planar Au electrodes. This includes spin coating of photoresist, patterns printing using a photolithography system, spin coating of SWCNTs, and lift off using acetone as presented in Steps 5–7. It’s worth mentioning that before SWCNTs deposition, oxygen plasma is performed to improve the adhesion between SWCNTs and the COC substrate.

**Figure 2:**
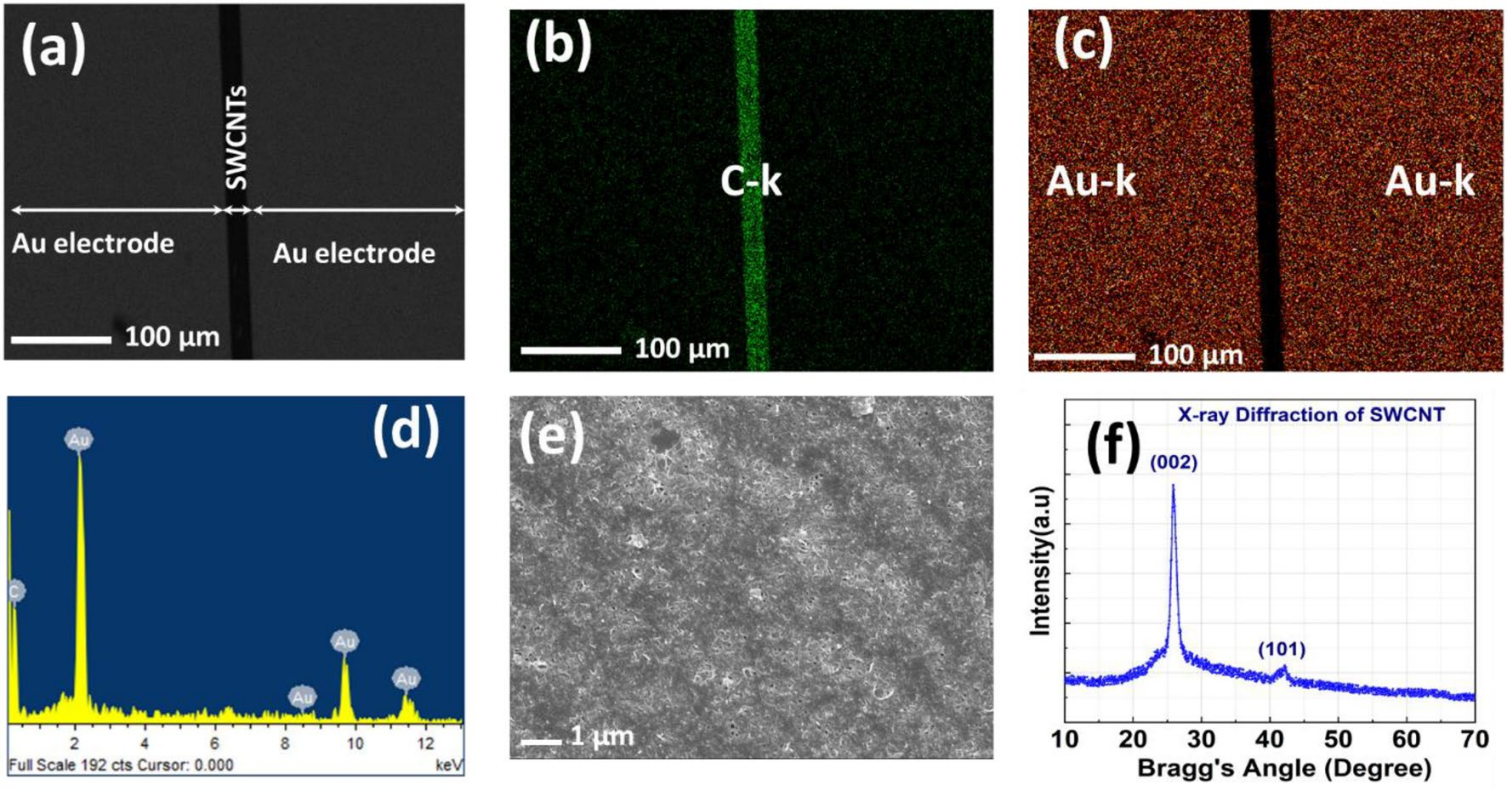
The Chemical and Physical characterization of the sample depicting (**a**) the planar structure of an Au-SWCNTs-Au-based device, (**b**) and (**c**) the color mapping of carbon (SWCNTs) and Au-electrodes, respectively, (**d**) the energy dispersion spectroscopy (EDS), (**e**) SEM image of the deposited SWCNTs film. (**f**) The interplanar spacing of the SWCNTs using XRD.

Figure 2 shows the physical characteristics of the fabricated device using SEM micrographs, the corresponding color mapping of the elements in a planar device, and X-ray diffraction studies of the SWCNTs thin film used to extract all the device characteristics. Figure 2a depicts the planar structure of an Au-SWCNTs-Au-based device with a 20 µm width, and 2–10 nm thickness, of SWCNTs as a switching medium. Figures 2b and c show the color mapping of carbon (SWCNTs) and Au-electrode, respectively, confirming the lateral sandwiched SWCNTs film between the Au electrodes. The energy dispersion spectroscopy (EDS) in Fig. 2d demonstrates the presence of C and Au peaks in the device.

The interatomic spacing of the graphitic lattice, which is correlated with the nanotube's diameter, is determined with the help of the X-RAY DIFFRACTION (XRD) technique, as shown in Fig. 2f. The two main XRD peaks at (002) and (100) in the XRD pattern of SWCNTs are observed. The (002) peak normally rises at a height of about 26°, whereas the (100) peak rises at a height of around 44°. These peaks reflect the hexagonal lattice structure of SWCNTs. The XRD pattern of SWCNTs may also show additional peaks that correlate to higher-order diffraction in addition to the (002) and (100) peaks, but we do not observe any additional peak, indicating highly pure SWCNTs used to fabricate the memristor device.

## Results and discussion

**Figure 3:**
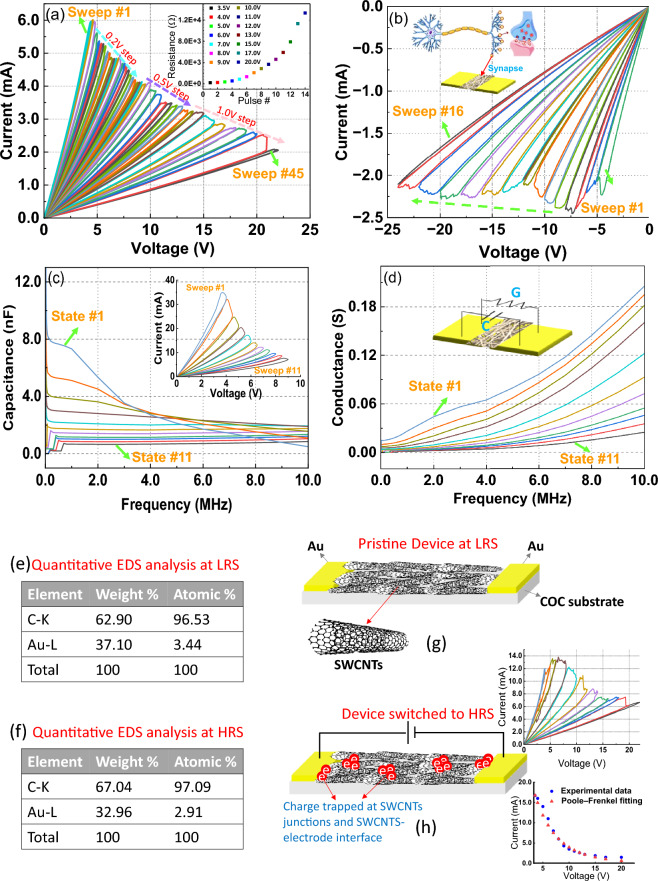
Device electrical characterization to confirm the analog switching behavior for the device resistance and capacitance. (**a**) 45 consecutive I–V sweeps from 4 to 22 V, the inset shows the multistate behavior under the application of consecutive threshold voltages. (**b**) 16 consecutive I–V sweeps from − 5 V to − 24 V. (**c**) and (**d**) represent the simultaneous Capacitance-Frequency and Conductance-Frequency measurements, respectively. Inset of (**c**) 11 consecutive I–V sweeps from 4 to 9 V. (**e**) and (**f**) Quantitative EDS analysis at LRS and HRS, respectively. (**g**) and (**h**) schematics to demonstrate the switching mechanism of the device. (h)-insets: I–V sweeps of the device after depositing new SWCNTs layer to retrieve the switching ability of the device, fitting results between the multistate switching characteristic presented by the SWCNTs-based memristor and Poole–Frenkel model. Drawings are prepared using Mindthegraph software and PowerPoint drawing tools.

To explore the electrical characteristics of the device, Keithley SCS-420 Source Measure Unit (SMU) is used utilizing voltage sweeping and pulse modes. As shown in Fig. 3a, 45 consecutive dual sweep voltages are applied, with stopping voltages ranging from 4 to 22 V. Two main observations can be revealed here to reflect the potential characteristics of the fabricated device for energy-efficient bio-inspired computation. First, a pristine device starts at a low resistance state, and a higher resistance is achieved by applying a voltage sweep with a higher stopping voltage, which reflects fully analog switching behavior. Second, the fabricated device exhibits long-term memory and no overlap takes place between the different resistance states. Figure 3a-inset shows the multistate behavior under the application of consecutive threshold voltages. Figure 3b exhibits a unipolar switching behavior as the device switches from a low resistance state (LRS) to a high resistance state (HRS) with the application of 15 consecutive dual voltage sweeps with stopping voltages starting from − 5 V to − 25 V. It is important to notice from Fig. 3a and b that the device remembers its last state regardless of the sweep polarity. This is mainly associated with the symmetric electrodes in the device structure. The initial resistant state of the device has been obtained via careful optimization of the SWCNTs solution. To elaborate, the concentration of SWCNTs is increased gradually in the DI solution until a continuously connected SWCNTs monolayer is achieved as presented in Fig. 2e. This leads to an initial low resistance state in the fabricated memristor device. For the lower and higher SWCNTs concentrations, individually dispersed SWCNTs and junk of SWCNTs are observed, respectively, in SEM as revealed in Figure S1. The monolayer of the SWCNTs network is confirmed via the AC mode of the atomic force microscope. Figure S2 shows the topography image of the SWCNTs network, which is used as the switching layer in our device. As the diameter of the used SWCNTs is 10 nm^17^, the obtained height of 12.6 nm confirms that the switching medium used in our device is the monolayer of the SWCNTs network. To evaluate the reliability of the fabricated memristors. Devices are selected randomly and the yield is calculated based on the measurement results. In this work, a device is considered to be working if it is found initially in LRS and exhibits the ability to switch between LRS and HRS in an analog manner. The obtained yield (88%) can be improved via further optimization of the fabrication process^8,18^. The ability to simultaneously tune the capacitance of the memristor device fabricated in this work is investigated using C-V units (CVU), from Keithley SCS-420 by utilizing Frequency Sweep-DC Bias mode. As presented in Fig. 3c, capacitance (C)-frequency (F) curves are obtained at each new written resistance state (see Fig. 3c-inset)) at 2 V DC voltage bias. This voltage is less than the minimum writing voltage of the device to avoid disturbing the state of the device. As shown in Fig. 3, the memristor’s capacitance can be tuned concurrently with the resistance change of the device. This reflects memimpedance behavior which has great value in many emerging applications; such as tunable RF components, and computation-in-memory (CIM)^19,20^. Figure 3d presents the device’s conductance levels that are measured simultaneously with the device capacitance to confirm the ability to tune the mem-impedance of the fabricated memristor. The device exhibits two biological behaviors; synaptic plasticity and synaptic efficacy, which make it a great candidate to perform bio-inspired computation.

As explained, interestingly, the device’s resistance increases with every I-V sweep, and more deep analysis can be provided here to have an insight into the switching mechanism that takes place in the device. Joule heating has been reported to be responsible for the switching events (from LRS to HRS) occurring in several metal oxides-based memristor devices^9^. According to this explanation, once a certain power of heating is attained in the memristor, the conductive filaments are ruptured abruptly due to oxidation reactions. Moreover, the work reported in Ref.^21^ demonstrates that at a high current density in the air, CNTs interconnect breakdown occurs due to the etching of the CNTs outer shells caused by oxygen. To validate any possible oxidation of SWCNTs in the switching medium of the device, quantitative EDS analysis is conducted, as shown in Fig. 3e and f. From the obtained results, we conclude that there is no oxidation of the SWCNTs after multiple I-V sweeps; thus, Joule heating is not responsible for the memory effect of the memristor device reported in this work. Also, this can be evident by the obtained smooth (analog) switching. Instead, the charge trap phenomenon has been associated with high retention and long-term CNTs-based memory devices^22,23^. As depicted in Fig. 3g and h, when the switching voltage is applied between the planar electrodes, charge trapping occurs at the junctions between SWCNTs, and at the interface between CNTs and electrodes^24^, leading to screening the internal field^2,25^ and hence reducing the movement (drift velocity) of charge carriers, which results in increasing device resistance. To further prove this, SWCNTs are re-deposited on a fully switched device (at HRS), and as presented in Fig. 3 (h)-inset, the device retrieves its switching ability as the new SWCNTS have no trapped charges. Based on this explanation, the Poole–Frenkel model that describes charge tapping defects^26^ is used to fit the I-V data of the fabricated memristor. As shown in Fig. 3h-inset, the multistate switching characteristic presented by the SWCNTs-based device exhibits strong agreement with the Poole–Frenkel model. This confirms the charge-trapping phenomena being associated with the switching mechanism of the fabricated device. As provided in Table 1, the memristor device reported in this work has the superiority to provide analog switching and memimpedance features, compared to the CNTs-based memristor devices reported in the literature. The key memory characteristics of the device are utilized for energy-efficient and high-accuracy inference in SNN as detailed in the following section.

**Table 1:** Comparison between the memristor device presented in this work and the CNTs-based memristor devices reported in the literature.

Reference	Switching medium	SW*/MW** CNTs	Planar/vertical structure	Long-term memory	Analog switching	Mem-impedance
^12^	CNTs	MW	Vertical	–	NO	–
^14^	CNTs	MW	Vertical	–	NO	–
^16^	CNTs-SiO_2_	SW	Vertical	Yes	NO	–
^27^	CNTs	–	Vertical	–	NO	–
^15^	CNTs: GQD	MW	Vertical	Yes	NO	–
^11^	CNTs-Au nanoislands	MW	Planar	–	NO	–
^28^	CNTs	MW	Vertical	–	NO	–
This work	CNTs	SW	Planar	Yes	Yes	Yes

*SW: Single wall.

**MW: Multiwall.

– means this hasn’t been explored/reported.

### Device deployment in SNN

In this work, we utilize the highly analog-switching behavior and the long-term memory feature of the fabricated memristor device to implement (SNN). Details about the adopted spiking neuron model, SNN architecture, Device deployment in SNN, accuracy, and energy efficiency evaluation are detailed here.

**Figure 4:**
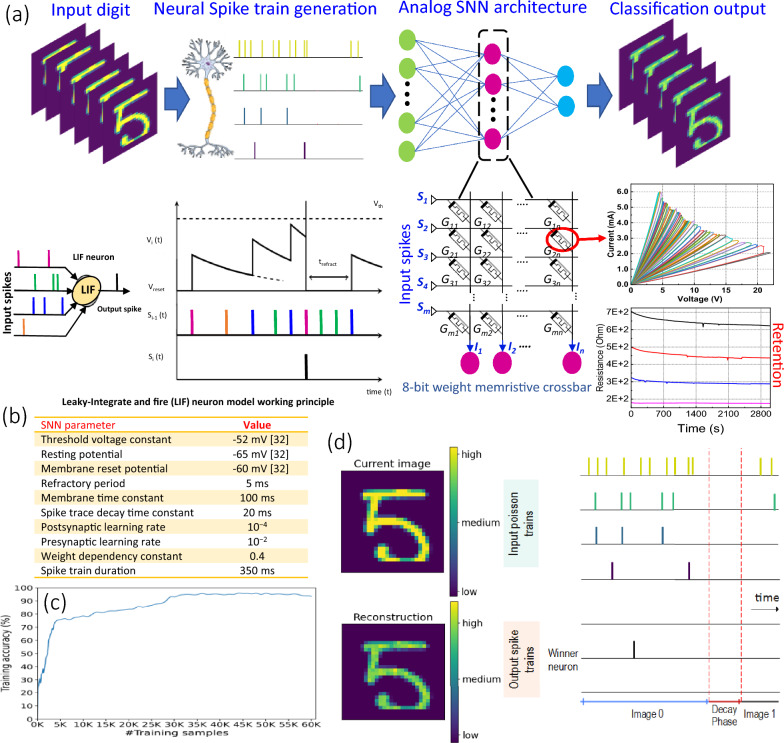
Memristor-based SNN implementation. (**a**) LIF neuron and its dynamics along with the 8-bit memristive crossbar with M wordlines (input voltages) and N bitlines, (**b**) SNN simulation setup. (**c**) Training accuracy vs. training samples for 20 epochs. (**d**) The schematic of the spike trains activity. Drawings are prepared using *Mindthegraph* software, *PowerPoint* drawing tools, and MNIST dataset^34^ images generated using *Matplotlib*.

The Leaky Integrate and Fire (LIF) neuron model presented in Ref.^29^ is adopted in this work as it provides a better trade-off between accurately mimicking biological neurons and the implementation simplicity^30,31^. The LIF model performs spatial and temporal integration of synaptic inputs to generate a spike when the membrane voltage reaches a certain threshold. After spiking, the neuron goes into a refractory period^30^. The LIF model can be expressed as shown in Eq. (1)^29^:

1 $${\tau_{mem}}\,\frac{\partial v\left( t \right)}{{\partial t}}\, = \, - v\left( t \right) + {v_{rest}} + I\left( t \right),$$

where *τ*_*mem*_ is the membrane time constant of the neuron, *v*(*t*) is the neuron membrane potential, *rest* is the resting membrane potential, and *I*(*t*) is the total current of a neuron at time *t*. Figure 4a shows a LIF neuron and its dynamics. As depicted, the LIF neuron receives spike inputs from multiple presynaptic neurons and accumulates the weighted spikes to increase its membrane potential. When the membrane potential reaches the threshold voltage *v*_*th*_, the neuron generates an output spike, decaying its membrane potential to a reset voltage *v*_*reset*_. Then, the neuron enters a refractory period in which it does not accumulate any incoming spikes. Once the refractory period has lapsed, the neuron will be active again to receive input spikes. When an active neuron receives no input spike, its membrane potential will gradually decay (leak).

To demonstrate the analog-based multi-bit potential of the device, a three-layer analog SNN architecture is developed. Thus, the analog SNN architecture uses 8-bit weight per single device to achieve high inference accuracy, while improving the density and energy efficiency. First, spike trains are generated from the input pixel vector using Poisson encoding schemes^32^. It should be noted that a pre-processing step is needed to convert the stimulus (two-dimensional input image) pixels into a one-dimensional (1D) vector of pixels. The 1D pixel vector is fed to the Poisson encoder that generates the corresponding spike train, which is equivalent to the input pixel intensity. The spike train is then fed to the analog SNN network built with LIF neurons. The LIF neurons in the analog SNN can use the multiply and accumulate the result of the analog crossbar directly, circumventing the need for costly Analog to analog-to-digital converters (ADCs). Thus, this analog nature of the SNN exploits the multi-bit storage capability of the device for energy-efficient operation.

The analog switching characteristic of the memristor device enables the user to store multibit weights in a single device. In this particular architecture, a memristive device stores an 8-bit weight. Therefore, the memristive devices are structured in a crossbar array to build a CIM unit, where computation and storage are integrated within the same physical location. The 8-bit memristive crossbar shown in Fig. 4a with *M* wordlines (input voltages) and *N* bitlines, in which the wordlines and bitlines are connected through a memristive bit-cell (1T1R) at their intersection. The crossbar can perform Vector Matrix Multiplication (VMM) operation by applying a voltage vector *V* = *V*_*i*_ (where *i* ∈ {1*,M*}) to the crossbar matrix of conductance values *G* = *G*_*ij*_ (where *i* ∈ {1*,M*}, *j* ∈ {1*,N*}). At any instance, each column can perform a multiplication-and-accumulation (MAC) operation, with the output current vector *I*, in which each element of the output current is obtained as shown in Eq. (2).

2 $${I_j} = \sum\limits_{i = 1}^M {V_i} \cdot {G_{ij}}.$$

All *N* MAC operations are performed with O(1) complexity, which is essential to implement energy-efficient SNN. The simulation setup used for the analog SNN implementation and evaluation is presented in Fig. 4b^33^. The SNN is implemented and trained in Python using bindsnet^34^, pytorch, and NVIDIA CUDA libraries. The MNIST dataset^35^ is used to train and evaluate the accuracy of the network. The MNIST image pixels are converted into spike trains using the Poisson encoding scheme, where each spike train is 350 ms in length (duration).

The training progress of the network is evaluated by gradually increasing the number of training samples every 20 epochs. Figure 4c shows the evolution of the training accuracy as a function of training samples. As presented, the training accuracy reaches its peak accuracy, 95%, when trained with 44,000 samples. Afterward, the network stabilizes with minor over-fitting. To evaluate the inference accuracy of the architecture, the input image pixels are first converted into spike trains as shown in Fig. 4d. From the figure, it can be observed that the spike trains are dependent on the pixel intensity and they are divided into three main levels. High-intensity image pixels are converted into high-rate spike trains (yellow). Medium-intensity pixels, e.g., pixels around the edges of the digit, are converted into medium-rate spike trains (green, blue). Finally, low-intensity pixels are converted into low-rate spike trains (purple). The figure also shows the reconstructed image from the inference output spikes of the network. The inference accuracy evaluation shows that the SNN achieves 95% inference accuracy when provided random image from the test dataset of the MNIST dataset. To evaluate the energy consumption of the CIM-based analog SNN architecture, the trained 8-bit weights of the network are programmed to the memristive crossbar, and the spike trains are provided as input for the VMM operation on the crossbar. To determine the inference energy efficiency of the device crossbar-based SNN, first, we extract the average spike activity of the network from behavioral simulation. Then, the write and read energy of the memristor device is extracted from device-level characterization by voltage sweeping which gives the current across the memristor at different resistance levels. The current and voltage pairs are then used to determine the write and read energy of the memristor analytically. Finally, the memristor writes and reads energy values along with the behavioral-level spike activity of the network are used to determine the energy efficiency. Results show that the proposed CIM implementation performs VMM in an energy-efficient manner with few femtojoules per spike. This demonstrates the potential of the fabricated multi-bit memristive device to realize energy-efficient SNN implementation for edge computing.

## Conclusion

This paper presented a SWCNTs-based memristor device using a monolayer of SWCNTs. The key insights of the fabricated device are the analog switching ability along with its long-term memory which provide a great opportunity to achieve compact neuromorphic devices. The pivotal memory characteristics of the device were exploited to implement SNN and perform classification in a high accuracy and energy-efficient manner. The results reported in this work are considered a milestone towards utilizing environmentally and economically sustainable materials to build emerging memory devices for energy-efficient computation at the edge.

### Supplementary Information

Supplementary Figures.

## Data Availability

The data that support the findings of this study are available from the corresponding author upon reasonable request.

## References

[1] *In-memory computing for deep learning and beyond*. [18-4-2023]. https://www.mpi-halle.mpg.de/541049/in-memory-computing-for-deep-learning-and-beyond.

[2] Jašinskas V (2020). Electronic and ionic electric field screening and persistent built-in electric field in carbon nanotube/PCBM films. Physica Status Solidi.

[3] Boybat I (2018). Neuromorphic computing with multi-memristive synapses. Nat. Commun..

[4] Abbas Y (2020). Improved figures of merit of nano-Schottky diode by embedding and characterizing individual gold nanoparticles on n-Si substrates. Nanotechnology.

[5] Tizani L (2021). Single wall carbon nanotube based optical rectenna. RSC Adv..

[6] Yang CC, Li S (2007). Investigation of cohesive energy effects on size-dependent physical and chemical properties of nanocrystals. Phys. Rev. B.

[7] John RA (2022). Reconfigurable halide perovskite nanocrystal memristors for neuromorphic computing. Nat. Commun..

[8] Huang Y (2023). Reliability improvement and effective switching layer model of thin-film MoS2 memristors. Adv. Funct. Mater..

[9] Mohammad B (2016). State of the art of metal oxide memristor devices. Nanotechnol. Rev..

[10] Chaim A (2022). PrMem: Novel flexible biodegradable paper-graphene oxide-based memristor. MRS Bull..

[11] Radoi A, Dragoman M, Dragoman D (2011). Memristor device based on carbon nanotubes decorated with gold nanoislands. Appl. Phys. Lett..

[12] Il'ina MV (2022). Memristors based on strained multi-walled carbon nanotubes. Diamond Relat. Mater..

[13] Singh RK, Kumar R, Singh DP (2016). Graphene oxide: Strategies for synthesis, reduction and frontier applications. Rsc Adv..

[14] Il'ina MV (2017). Memristive switching mechanism of vertically aligned carbon nanotubes. Carbon.

[15] Wang L (2021). Dual-tunable memristor based on carbon nanotubes and graphene quantum dots. Nanomaterials.

[16] Min J-G, Cho W-J (2021). Chitosan-based flexible memristors with embedded carbon nanotubes for neuromorphic electronics. Micromachines.

[17] Abbas Y (2022). Focused ion beam engineering of carbon nanotubes for optical rectenna applications. ACS Appl. Nano Mater..

[18] Lin, Y.-C., et al., *Recent Advances in 2D Material Theory, Synthesis, Properties, and Applications.* ACS nano, 2023. 10.1021/acsnano.2c12759 PMC10324635 37219929

[19] Wakrim T (2016). From MEMRISTOR to MEMImpedance device. Appl. Phys. Lett..

[20] Kilani D (2021). C3PU: Cross-coupling capacitor processing unit using analog-mixed signal for AI inference. IEEE Access.

[21] Santini C (2011). A study of Joule heating-induced breakdown of carbon nanotube interconnects. Nanotechnology.

[22] Chen Y (2014). Polymer memristor for information storage and neuromorphic applications. Mater. Horizons.

[23] Li L, Wen D (2018). Memristic characteristics from bistable to tristable memory with controllable charge trap carbon nanotubes. Nanomaterials.

[24] Rezk A (2020). Charging and discharging characteristics of a single gold nanoparticle embedded in Al2O3 thin films. Appl. Phys. Lett..

[25] Cai D, Liu L (2013). The screening effects of carbon nanotube arrays and its field emission optimum density. AIP Adv..

[26] Chang Y-F (2014). Intrinsic SiOx-based unipolar resistive switching memory II Thermal effects on charge transport and characterization of multilevel programing. J. Appl. Phys..

[27] Veksler D, Veksler D (2020). Memory update characteristics of carbon nanotube memristors (NRAM®) under circuitry-relevant operation conditions. 2020 IEEE International Reliability Physics Symposium (IRPS).

[28] Il’ina MV (2020). Dependence of the memristor effect of carbon nanotube bundles on the pressing force. Fullerenes Nanotubes Carbon Nanostruct..

[29] Gerstner W (2014). Neuronal Dynamics: From Single Neurons to Networks and Models of Cognition.

[30] Zare M, Zafarkhah E, Anzabi-Nezhad NS (2021). An area and energy efficient LIF neuron model with spike frequency adaptation mechanism. Neurocomputing.

[31] Fang X (2022). Memristive lif spiking neuron model and its application in morse code. Front. Neurosci..

[32] Banerjee, D., et al. *Efficient optimized spike encoding of multivariate time-series*. In: *Neuro-Inspired Computational Elements Conference*. 2022.

[33] Platkiewicz J, Brette R (2010). A threshold equation for action potential initiation. PLoS Computat. Biol..

[34] Hazan H (2018). Bindsnet: A machine learning-oriented spiking neural networks library in python. Front. Neuroinform..

[35] LeCun, Y., *The MNIST database of handwritten digits.* http://yann.lecun.com/exdb/mnist/, 1998.

