# Supplementary material for: Analog monolayer SWCNTs-based memristive 2D structure for energy-efficient deep learning in spiking neural networks
Source: Sci Rep. 2023 Dec 4;13:21350. doi: 10.1038/s41598-023-48529-z (PMC10696067; doi:10.1038/s41598-023-48529-z)
Supplement: Supplementary file 1 — Supplementary Figures. [file 41598_2023_48529_MOESM1_ESM.pdf]

To obtain the optimized low-resistance monolayer of SWCNTs on the COC substrate, we prepared different solutions with different concentrations of SWCNTs in DI water. After careful SEM analysis, we observed three different SWCNTs morphologies in SEM micrographs, as shown below. Figure (a) and (b) show the junks of CNTs and individually dispersed CNTs, respectively, whereas Figure (c) depict the optimized and minimal thickness of SWCNTs, which we considered as the monolayer. We considered this lowest possible thick CNT film as a monolayer for the memristor applications for neuromorphic computing.

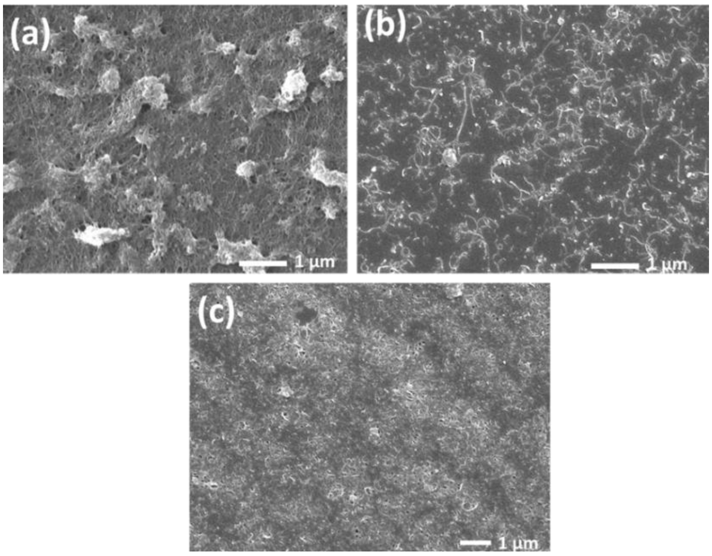

Figure S1: The SEM micrographs of SWCNT films on COC substrate showing (a) the junk of SWCNTs, (b) individually dispersed SWCNTs, and (c) the monolayer of SWCNTs.

To confirm the monolayer of the SWCNTs network, we carried out the AC mode of the atomic force microscope. Figure S2 shows the topography image of the SWCNTs network, which is used as the switching layer in our device. Figure S2 (a) exhibits the topography of the monolayer of the SWCNTs network, whereas Figure S2 (b) confirms the height profile of the monolayer layer along the line drawn in Figure S (a). The height of 12.6 nm confirms that the switching medium used in our device is the monolayer of the SWCNTs network.

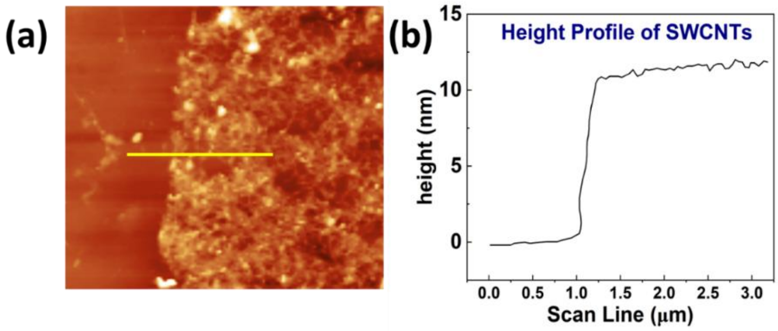

Figure S2: The atomic force microscope analysis of the SWCTNs network depicts (a) a well-connected network of SWCNTs and (b) the height profile of 12.6 nm drawn across the yellow line in Figure (a) confirming the monolayer of the CNTs network.
